# An alternative material for an effective treatment technique proposal in the light of bibliometric profile of global scientific research on antibiotic resistance and *Escherichia coli*

**DOI:** 10.1007/s10661-020-08678-4

**Published:** 2020-10-20

**Authors:** Semanur Şahin, Isil Akpinar, Nüket Sivri

**Affiliations:** 1 Department of Genetics and Bioengineering, Faculty of Engineering And Architecture, Nisantasi University, 34398 Istanbul, Turkey; 2 Department of Chemical and Environmental Engineering, University of Nottingham, Nottingham, NG7 2RD United Kingdom; 3 Department of Environmental Engineering, Faculty of Engineering, Istanbul University-Cerrahpasa, 34320 Istanbul, Turkey

**Keywords:** Water quality, Indicator Bacteria, Network visualization, Publication analysis, Metal-organic frameworks

## Abstract

Antibiotic resistance is considered by the countries to be a global health issue and a huge threat to public health. The reduction of resistant microorganisms from water/wastewater is of importance in environmental sciences since they are resistant in the aquatic environment. In this study, a bibliometric analysis of literature from the field of environmental science in water ecosystems from 2015 to 2019 was carried out using the keywords “Antibiotic Resistance (AR)” and “*Escherichia coli*”. Furthermore, using the keywords of “Fresh Water,” “Sea Water,” and “Waste Water,” 155, 52, and 57 studies were discovered, respectively. It is found that 217 studies of the total 2115 studies investigated on AR are mostly performed in the “Waste Water” by considering human health. Given the studies, an up-to-date solution should be proposed since the release of antibiotic-resistant bacteria (ARB) and antibiotic resistance genes (ARGs) from wastewater treatment plants needs to be mitigated. For this reason, it is obvious that working on micro and macro ecosystems will increase the probability of solutions in antibiotic resistance. A discussion of removal techniques for coliform bacteria, particularly antibiotic resistant *Escherichia coli*, was presented. One of the unique values of this study is to offer an innovative solution that removing them by metal-organic frameworks (MOFs) are emerging crystalline hybrid materials. MOFs are used for environmental, biological, and food antimicrobial substances efficiently. Therefore, we can give inspiration to the future studies of antimicrobial resistance removal via adsorption using MOFs as adsorbents.

**Figure:**
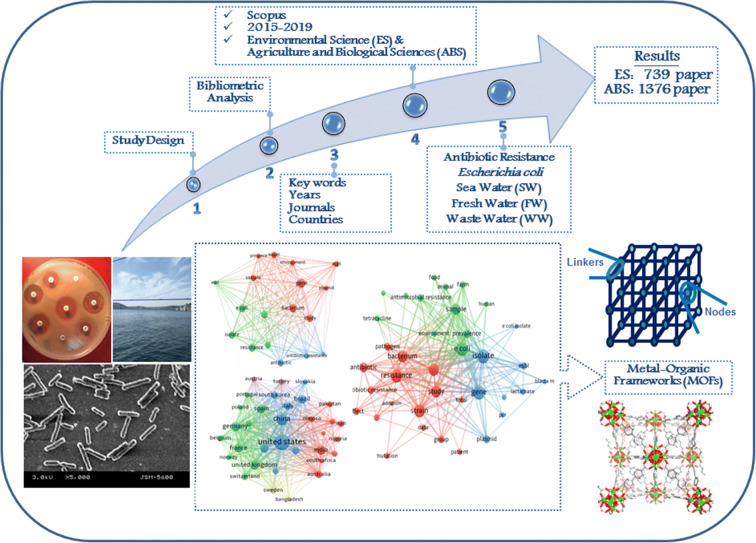
Graphical Abstract

Graphical Abstract

## Introduction

Antibiotic resistance (AR), antibiotic-resistant bacteria (ARB), and antibiotic resistance genes (ARGs) are entering into the aquatic environment by means of anthropogenic activities including the discharge of feces and from water and wastewater plants. Wastewater and wastewater treatment plants are regarded as a source of antibiotic resistance. Wastewater has fecal residues including antibiotic resistance bacteria, antibiotic resistance genes, metals, organic pollutants, and the residue of antibiotics (Thakali et al. 2020; Voigt et al. 2020). In addition, these bacteria are able to spread their genes into water-indigenous microbes, which also contain resistance genes (Baquero et al. 2008). They are not completely mitigated during treatment processes in sewage treatment plants; therefore, they can be distributed to the aquatic environment (Michael et al. 2013; Rizzo et al. 2013; Hembach et al. 2019; Voigt et al. 2020). Given this spread of ARGs in surface water and insufficient degradation in sewage treatment plants, antibiotic resistance poses a great threat to public health and to ecology (Krzeminski et al. 2020; Thakali et al. 2020; Voigt et al. 2020). Furthermore, the World Health Organization (WHO), the United Nations (UN), and the European Union consider antimicrobial resistance (AMR) as a global public health threat (World Health Organization 2015; United Nations 2016; Krzeminski et al. 2020).

Recently, studies on antibiotic resistance in water has gained significant attention, thus, our knowledge of antibiotic resistance has improved. However, no bibliometric analysis of studies on AR has been carried out in the environmental sciences. For this purpose, we used bibliometric analysis to analyze published articles in terms of quantity and quality using mathematical methods. This analysis is an important and common method to assess research activity on a certain topic. Additionally, in bibliometric analysis, information on publications, the most productive countries/institutes, and authors in the prominent journals and the quartile of categories, which are the criteria of the scientific effects of journals, are presented (Ellegaard and Wallin 2015). Despite all this data, unfortunately, there are still limited studies on antibiotic resistance and solutions that are developed for the removal of antibiotic resistance.

*Escherichia coli* is the most frequently investigated microorganism in relation to the conducted AR studies in aquatic environment as it is known that there is a continuous monitoring for *Escherichia coli* to determine the bacteriological quality of water. In many studies on sanitation of natural waters, the presence of *Escherichia coli* is investigated since *Escherichia coli* appears to be the best indicator of bacteriological quality of water owing to the availability of affordable, fast, sensitive, specific, and easier detection methods. It is also a particular indicator to be used rather in fecal contamination than fecal coliforms because the test is used for the determination of fecal coliforms and to identify non-fecal coliform bacteria (Odonkor and Ampofo 2013).

In this study, the bibliometric analysis of global research trends across studies on “antibiotic resistance” and “*Escherichia coli*” was evaluated from 2015 to 2019 and a total of 2115 studies were investigated. The studies using the keywords of “Antibiotic Resistance” and “*Escherichia coli*” related issues in the most studied water ecosystems aquatic that are published in two research fields of “Agriculture and Biological Sciences” and “Environmental Science” were identified in worldwide. Also, in line with AR studies, we discussed the effective solutions to the antimicrobial resistance treatment before releasing into the aquatic environment as they are known to have potential hazard to the public and aquatic environment.

## Methods

Particular keywords of “antibiotic resistance” and “*Escherichia coli*” were used to search in Scopus database. *Escherichia coli* is present in the intestine of human and animals and released into the environment in fecal material. As fecal matter is the main source for disease-causing agents in water, fecal bacteria are widely used as indicators of the contamination which can affect rivers, sea beaches, lakes, ground water, surface water, recreational water, and many diverse activities (Odonkor and Ampofo 2013; Price and Wildeboer 2017).

In this database, two fields “Agriculture and Biological Sciences” and “Environmental Science” were selected. Furthermore, each of the review articles was categorized as environment, natural waters and wastewater, antibiotic resistance (AR), the journal name, quartile in category of the journal, the year of publication, and the countries. “Antibiotic Resistance,” “*Escherichia coli*,” “sea water (SW),” “fresh water (FW),” and “waste water (WW)” were used as keywords. Appropriate bibliometric indicators and visualization maps were also used to present the quantitative and qualitative analysis of the retrieved data.

Data search and collection were obtained from Scopus database in these two fields of Agricultural and Biological Sciences and Environmental Science in this study. The terms for the systematic search strategy included the title of the article, abstract, and keywords. Furthermore, the “document type” was not limited only to the articles. Therefore, the search was obtained with using the following criteria including the title of “antibiotic resistance” and “*Escherichia coli*” and publication year of (PUBYEAR, 2015), (PUBYEAR, 2016), (PUBYEAR, 2017), (PUBYEAR, 2018), (PUBYEAR, 2019) and the subject areas of Agricultural and Biological Sciences and Environmental Science.

The information retrieved from the Scopus database consisted of country names, antibiotic names (i.e., the paper if more than five drugs are studied, these were named as multidrug), working environment (i.e., FW, SW, WW), the name of the journals, quartile of category, and other information.

Several constraints were also identified in this study. First, the search was performed using antibiotic resistance and *Escherichia coli* in the abstract or title. The articles that do not comply with this constraint were identified as those that do not contain either *Escherichia coli* or Antibiotic resistance, or neither. Resistance may be studied in various topics; however, this study mainly focused on antibiotic resistance studies. “Antibiotic resistance,” “antimicrobial resistance,” and “bacterial resistance” were the selected keywords; however, the studies of “antibiotic sensitivity” were not included in this search. Moreover, the keywords of “coliform” and “Enterobacteriaceae” for *Escherichia coli* were accepted while searching the studies. The other constraint was identified as the environment where the samples were collected.

This study examined and analyzed the publications that were studied in the aquatic environment. Water environment was categorized as “freshwater,” “wastewater,” “seawater,” and different water environment was named as “water.” The keywords for “freshwater” were used as river, watershed, lake, waterways, groundwater, tap water, drinking water, and surface water depending on the region where it was taken from. The keywords for “wastewater” were used as “sewage, sludge, environmental water, recycled water, drainage water, reclaimed water”. The accepted keywords for “seawater” were “coastal, beach, marine, stream water, recreational water” and “surface water” depending on the region where it was taken from. On the other hand, the publications that do not contain water media were categorized as animal, plant, human, soil, illness, food, and feces. The soil category was taken into consideration when the soil was in contact with water, which was identified in non-environmental studies and was categorized as “E (none).” The other important constraint was that the location of the studies performed as a country. If none were mentioned in the abstract or title, this means that it was presented based on the country of the first author or corresponding author. Turkey as a word refers to not only a country but also an animal name, and this was a situation that can lead to a disagreement in country names; for this reason, an individual category of animals was created in order to eliminate the incomprehensibility.

In this analysis, original researches were particularly chosen even though the international presentations and book chapters were also found in the search. When it comes to the names of drugs, if there were less than five antibiotics subjected to study, their names were expressed as Antibiotic1, Antibiotic2, Antibiotic3, Antibiotic4, and Antibiotic5. However, in the case of having more than five antibiotics studied, “Multidrug” was used to identify the differences. On the other hand, if an antibiotic name was not included in the abstract or title but it was mentioned in the study, this antibiotic was accepted as “multidrug.” In the case of not containing an antibiotic name in the summary or title but containing a resistance gene, antibiotic name was included.

## Results and discussion

In the first search, with using the keywords of “Antibiotic Resistance” and “*Escherichia coli*,” 1376 and 739 studies in the field of “Agriculture and Biological Sciences” and “Environmental Science” were found to be published in 2015, 2016, 2017, 2018, and 2019 within the criteria investigated in the Scopus database until the date of 20th April, 2020, respectively.

In the case of adding “sea water” as an additional keyword into our search, 52 (23 studies in Agriculture and Biological Sciences and 29 studies in Environmental Science) more studies were found in the same subject. Furthermore, with using the keywords of “fresh water,” and “waste water,” 155 (47 studies in Agriculture and Biological Sciences and 108 studies in Environmental Science) and 57 (11 studies in Agriculture and Biological Sciences and 46 studies Environmental Science) studies were discovered, respectively; therefore, “wastewater” is considered the most common working environment for the study of “antibiotic resistance” and “*Escherichia coli*.”

Table 1 represents the most relevant journal names, publications, and quartile categories that contributed significantly to the studies of antibiotic resistance and *Escherichia coli* in the working environment of wastewater, seawater, freshwater, and water. It is observed that the quartile of categories changed to 1 and 2 in Table 1. It is found that the journal of “Science of the Total Environment” was ranked as the first journal among the first 10 journals in the field of Environmental Science studied in water studies from 2015 to 2019, and the journal of “Plos One” was ranked as the foremost journal in the field of Agriculture and Biological Sciences.

**Table 1:** The top 10 journals for antibiotic resistance and *Escherichia coli* studies on water environment published from 2015 to 2019. (Subject area: Agricultural and Biological Sciences and Environmental Science. QC: quartile of category and NoP: no of publications)

Rank	Environmental Science			Agricultural and Biological Sciences		
	Name of Journal	QC	NoP	Name of Journal	QC	NoP
1	Science of the Total Environment	Q1	39	Plos One	Q1	22
2	Water Research	Q1	22	Applied and Environmental Microbiology	Q1	11
3	Environmental Science and Technology	Q1	21	International Journal of Food Microbiology	Q1	6
4	International Journal of Environmental Research and Public Health	Q2	20	Water (Switzerland)	Q1	6
5	Environmental Science and Pollution Research	Q1	17	Journal of Food Protection	Q2	5
6	Environmental Monitoring and Assessment	Q2	12	International Journal of Environmental Science and Technology	Q2	4
7	Environmental Pollution	Q1	11	Marine Pollution Bulletin	Q1	4
8	Chemosphere	Q1	10	Environmental Microbiology Reports	Q1	3
9	Journal of Water and Health	Q2	10	Journal of Wildlife Diseases	Q2	3
10	Applied and environmental microbiology	Q1	9	Microbial Ecology	Q1	3

**Fig. 1:**
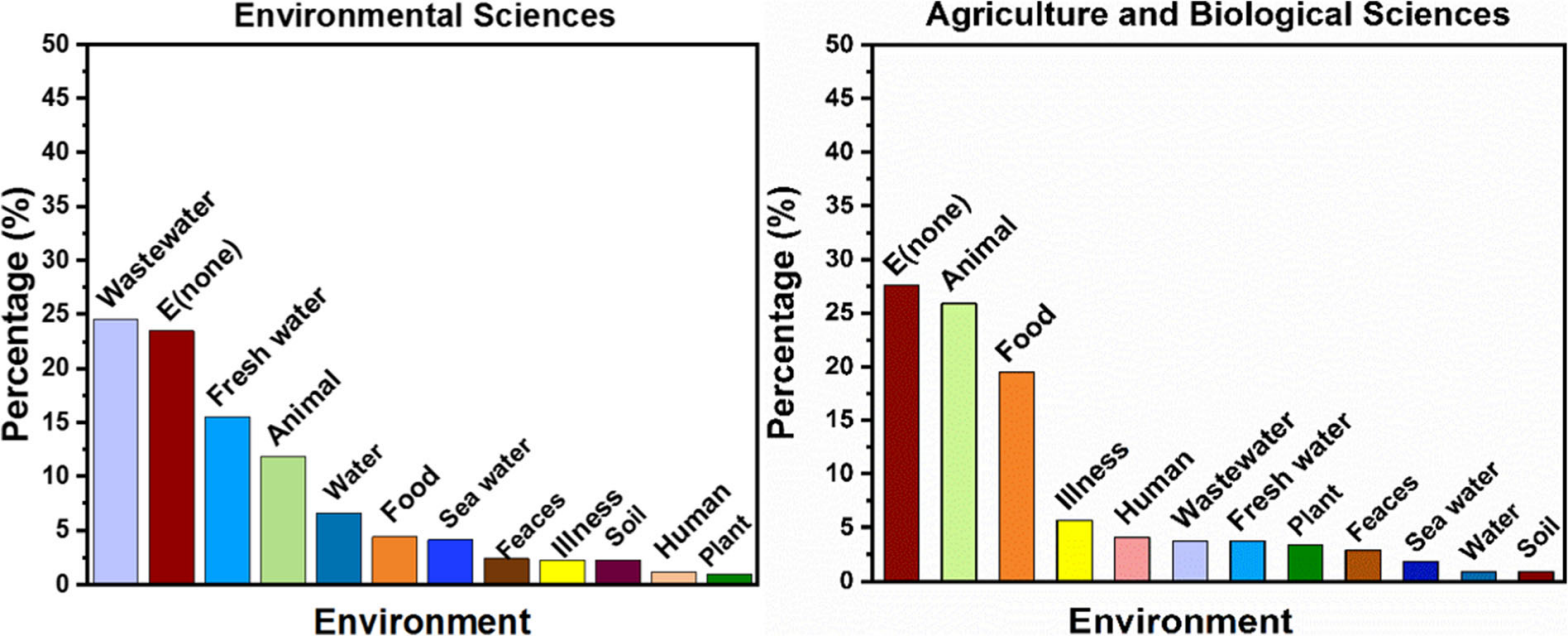
Comparison of antibiotic resistance and *Escherichia coli* studies that were carried out in different categories in the field of Agriculture and Biological Sciences and Environmental Science from 2015 to 2019 (E (none): non-environmental studies)

Figure 1 represents the number of studies on antibiotics resistance and *Escherichia coli* studies in various categories and areas. It is clearly seen that the water studies were significantly performed in Environmental Science (Fig. 1). Moreover, the categories of animal and food were highly observed in Agriculture and Biological Sciences. A decrease in the animal and food categories was revealed in the direction of W, WW, FW, and SW.

Based on this analysis, it can be said that antibiotic resistance was mostly studied in wastewater. *Escherichia coli* is the most commonly studied microorganism since *Escherichia coli* is regarded an indicator. The studies in the field of Agriculture and Biological Sciences and Environmental Science in the various categories with their rates as percentage were given. According to these studies in Environmental Science, the first five categories were found to be of wastewater (24.53%), E (none) (23.52%), freshwater (15.58%), animal (11.83%), and water (6.64%). Other categories were included in the chart were found to be of food (4.47%), seawater (4.18%), faces (2.45%), illness (2.31%), soil (2.31%), human (1.15%), and plant (1.01%) (Fig. 1).

**Fig. 2:**
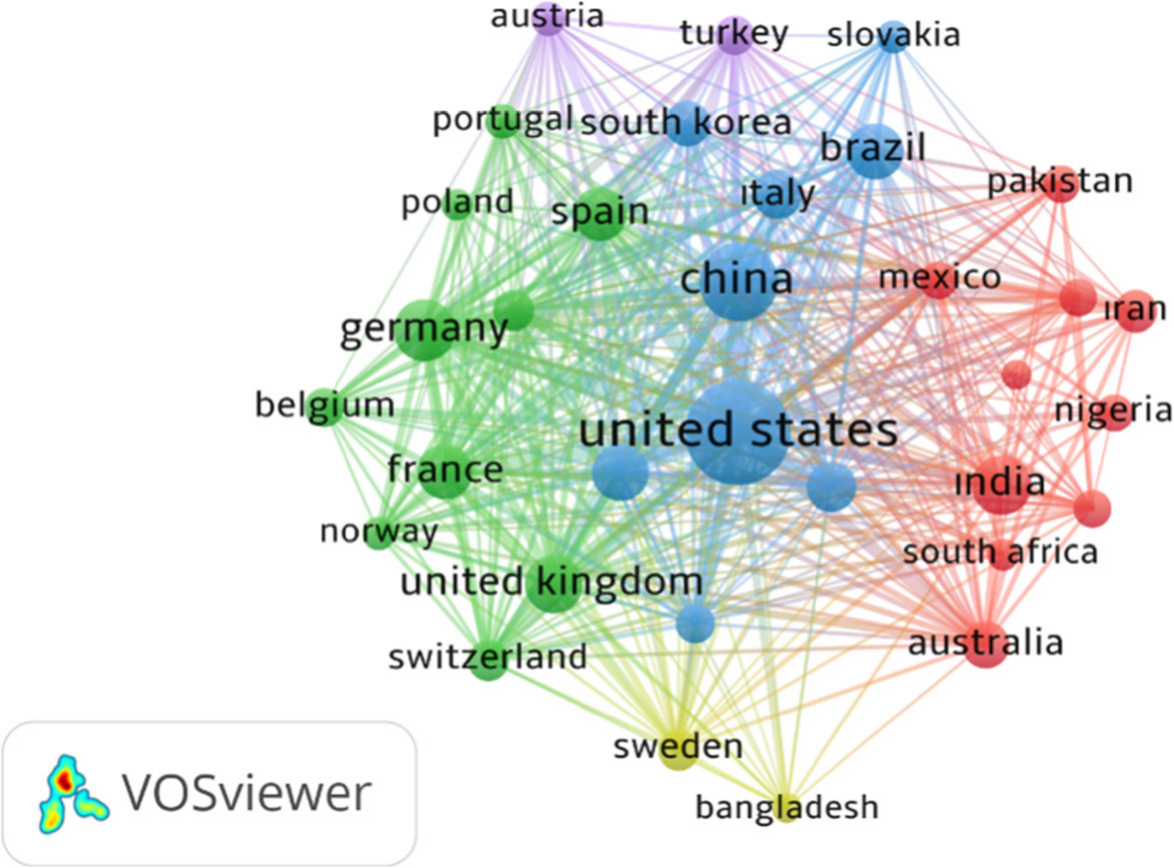
Network visualization map of countries for antibiotic resistance and *Escherichia coli* publications in the field of Agricultural and Biological Sciences from 2015 to 2019 (The countries were found less than 15 times in the 1376 studies were not added into the list)

**Fig. 3:**
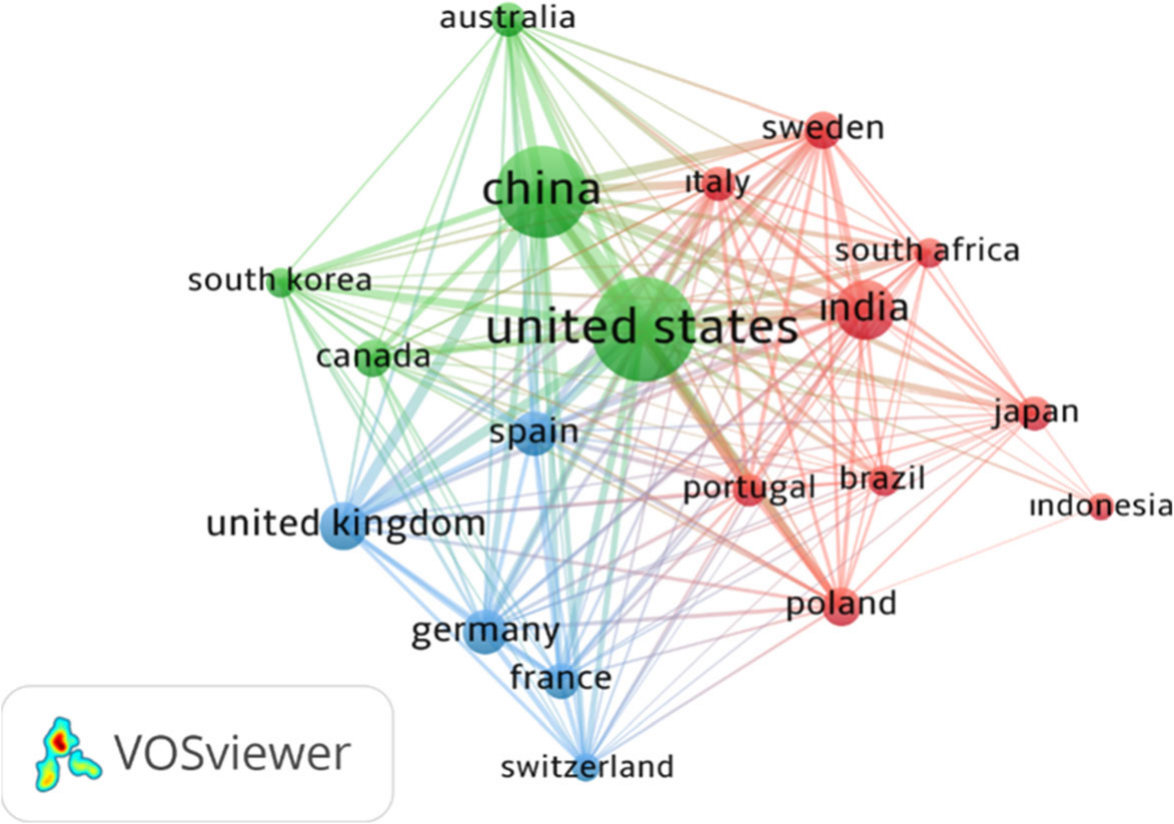
Network visualization map of countries for antibiotic resistance and *Escherichia coli* publications in the field of Environmental Science. Year: From 2015 to 2019. (The countries found less than 15 times in the 739 studies were not added into the list)

Figure 2 and Figure 3 present the country network visualization of publications on antibiotic resistance and *Escherichia coli* in Agricultural and Biological Sciences and Environmental Science from 2015 to 2019. We can identify the countries when the studies are frequently performed by using “Network visualization map”. The countries included in the figures that were found at least 15 times within the 1376 studies. USA and China are of the two countries lead the study of antibiotic resistance in water environment and *Escherichia coli* studies in both study fields. Egypt is the other country that we often encounter in these studies, following the USA and China. In bibliometric analysis, the countries can be identified based on the studies most frequently or less frequently performed. Network mapping provide information on which countries have less studies in terms of the field of the study. The sizes of the circles are proportional to the studies frequency.

To compare the studies in both fields, the leading six countries based on their number of publications on antibiotic resistance and *Escherichia coli* are given in Table 2. It is seen that the studies conducted in seawater in both fields were performed particularly in the USA. The studies performed in wastewater in the field of Environmental Science were found to be greater than that of the studies in other environments. In addition, the countries where the authors and affiliations are based given in Table 3 are found to be similar to the first 6 countries given in Table 2. Although Portugal was listed as the sixth country in the field of Environmental Science, Portugal was determined as the first on the list in terms of the authors and affiliations.

**Table 2:** The countries with having the highest number of publications on antibiotic resistance and *Escherichia coli*, particularly, in water environment from 2015 to 2019 in the field of Agricultural and Biological Sciences and Environmental Science

Environmental Science						Agricultural and Biological Sciences					
Country	SW	WW	FW	W	Total	Country	SW	WW	FW	W	Total
China	2	20	19	10	*51*	**United States**	3	6	13	2	***24***
United States	3	21	17	6	*47*	**China**	1	4	3	0	***8***
India	2	10	9	7	*28*	**India**	1	5	1	0	***7***
Brazil	1	9	6	1	*17*	**Nigeria**	0	3	2	1	***6***
Poland	0	7	6	2	*15*	**Bangladesh**	0	2	2	1	***5***
Portugal	2	11	1	1	*15*	**South Africa**	0	1	2	2	***5***

**Table 3:** Top-10 leading authors and affiliations in antibiotic resistance and *Escherichia coli* studies on water environment during the period from 2015 to 2019 (Subject area: Agricultural and Biological Sciences and Environmental Science)

Rank	Environmental Science			Agricultural and Biological Sciences		
	Author	Affiliation	NoP	Author	Affiliation	NoP
1	Manaia, C.M.	Universidade Católica Portuguesa, Porto, Portugal	11	Coutinho, H.D.M	Universidade Regional do Cariri, Crato, Brazil	12
2	Nunes, O.C	Universidade do Porto, Porto, Portugal	8	Schmidt, J.W	USDA ARS Roman L. Hruska U.S. Meat Animal Research Center, USA	9
3	Rizzo, L	Università di Salerno, Salerno, Italy	8	Arthur, T.M	USDA ARS Roman L. Hruska U.S. Meat Animal Research Center, USA	8
4	Fatta-Kassinos, D	University of Cyprus, Nicosia, Cyprus	7	Torres, C	Universidad de La Rioja, Logrono, Spain	8
5	Gu, A.Z	Cornell University, Ithaca, United States	7	Andremont, A	Hôpital Bichat-Claude-Bernard AP-HP, Paris, France	7
6	Heß, S	Helsingin Yliopisto, Helsinki, Finland	7	Johnson, J.R	University of Minnesota, Minneapolis, USA	7
7	Li, B	University of Science and Technology Beijing, Beijing, China	7	Scott, H.M	Texas A&M College of Veterinary Medicine & Biomedical Sciences, College Station, USA	7
8	Li, D	Fudan University, Shanghai, China	7	Shi, L	Jinan University, Guangzhou, China	7
9	Lundborg, C.S	Karolinska Institutet, Stockholm, Sweden	7	Tintino, S.R	Universidade Regional do Cariri, Crato, Brazil	7
10	Michael-Kordatou, I.	University of Cyprus, Nicosia, Cyprus	7	Wang, H	Sichuan University, Chengdu, China	7

**Fig. 4:**
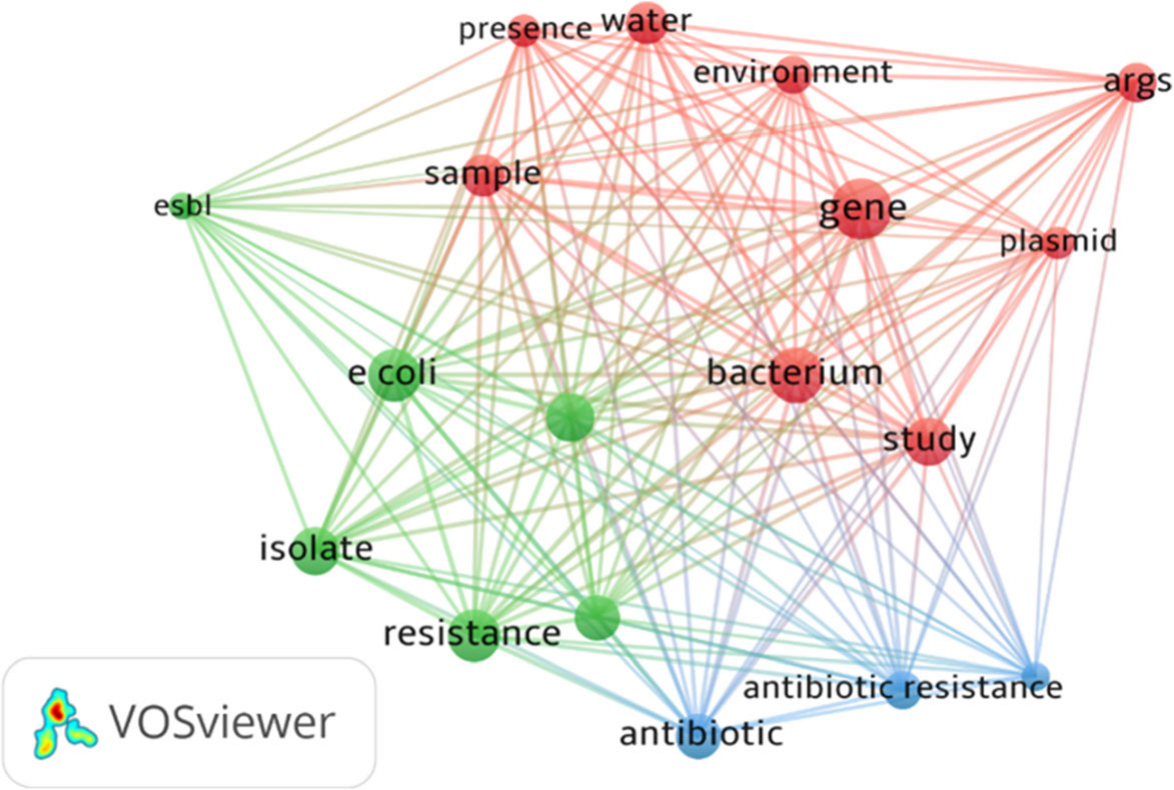
Research-topic network visualization for antibiotic resistance and *Escherichia coli* publications in the field of Environmental Science (2015–2019). (The minimum number of a keyword for the analysis is 150 occurrences, that is, the criterion is country names to appear at least 150 times among 739 retrieved studies)

**Fig. 5:**
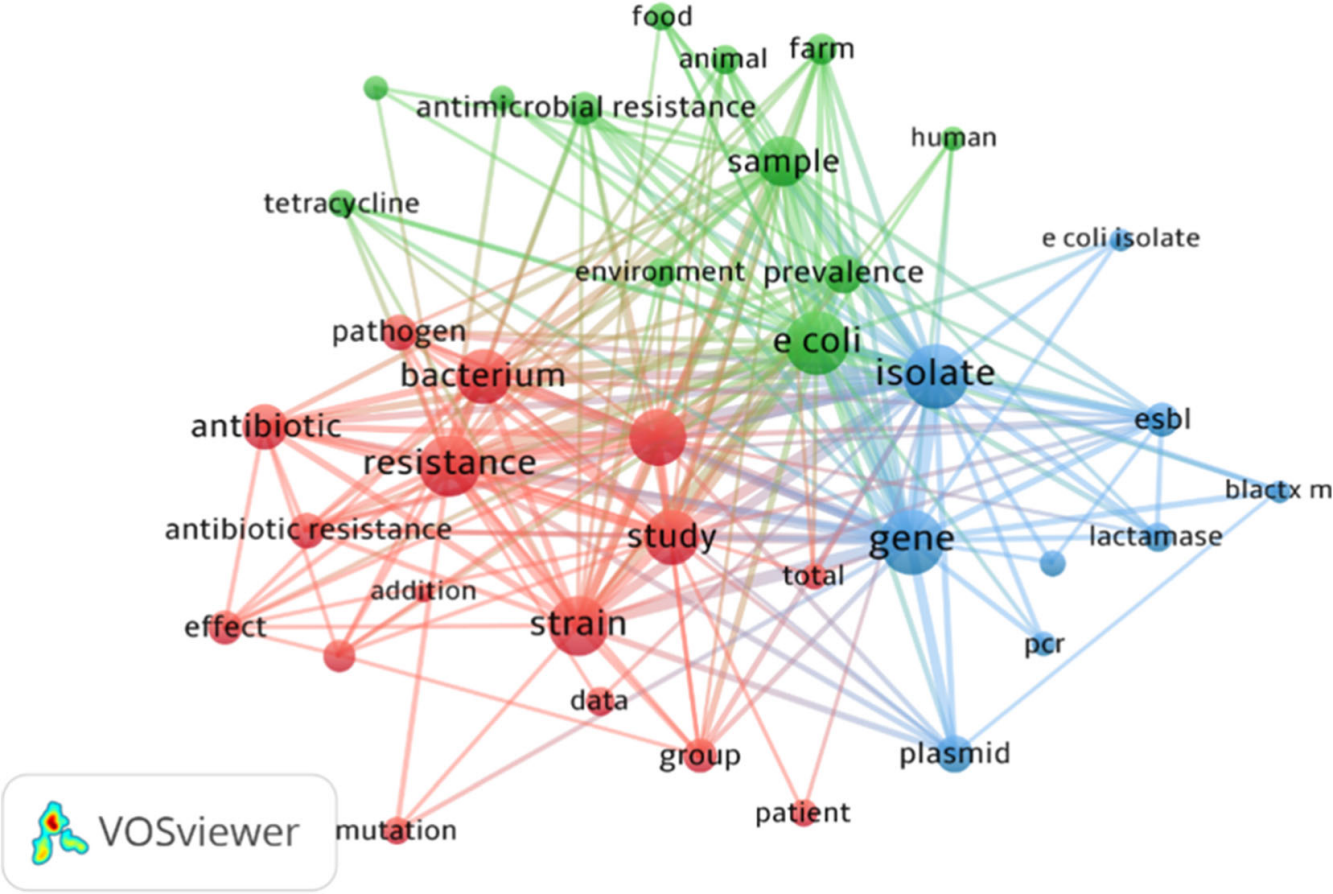
Research-topic network visualization for antibiotic resistance and *Escherichia coli* publications in the field of Agricultural and Biological Sciences (2015–2019). (Note: the minimum number of a keyword for the analysis is 10 occurrences, that is, the criterion is country names to appear at least 10 times among 1376 retrieved studies)

The network visualization having frequently repeated keywords in the studies on antibiotic resistance and *Escherichia coli* from 2015 to 2019 are given in Fig. 4 and Fig. 5. Figures 4 and 5 show the most frequently repeated terms and links with a minimum of 150 and 10 repetitions, respectively. The size of the circles and the number of connecting lines that contain the terms are expressed in direct proportion to the frequency of the keyword’s repetition. Similar keywords were encountered in both areas. In relation to the titles, the keyword “water” stands out differently in the field of “Environmental Science.”

The conclusion to be drawn here is that the use of keywords is not taken into consideration in the summary and title. This makes it difficult for the reader and/or researcher to perform more specific searches. Moreover, the researcher should be able to obtain the most basic information from the summary and title part without reading the entire study. Keywords should reflect the basics of the study in the best way, so that the researcher can easily obtain a preliminary idea. As a result of the examination, it is seen that a total of 114 studies from 739 articles, that is, 12% of these studies did not contain either one or both keywords. Moreover, the table shows that 59% of keywords did not consist of *Escherichia coli* in these 114 studies, suggesting that the keywords should be chosen more carefully.

Table 4 shows the proportions of one or both of keyword are being included in the summary or title in the articles found while creating the large data tables in the study. As we can see in Table 4, it was preferred to use similar keywords in the studies. The fact that these keywords are interconnected enables easy access to the publication while conducting research.

**Table 4:** The 10 keywords are the most found for antibiotic resistance and *Escherichia coli* studies in Agricultural and Biological Sciences and Environmental Science from 2015 to 2019

Rank	Agricultural and Biological Sciences			Environmental Science		
	Keyword	Occurrence	Relevance	Keyword	Occurrence	Relevance
1	*Escherichia coli*	923	0.01	Bacterium	988	0.59
2	Resistance	638	0.01	Gene	965	1.11
3	*E. coli*	619	0.08	*Escherichia coli*	701	0.26
4	Strain	456	0.07	Resistance	845	0.73
5	Gene	491	0.24	Isolate	692	0.99
6	Bacterium	433	0.16	Strain	631	0.62
7	Antibiotic Resistance	333	0.12	Antibiotic	618	0.57
8	Analysis	296	0.03	Water	55	0.79
9	Antimicrobial Resistance	273	0.23	Antibiotic Resistance	409	0.19
10	Environment	199	0.16	Antibiotic Resistance Gene	286	3.17

## Conclusion and future perspective

In this study, the results obtained are interpreted with an overview; thus, it is seen that in the literature, total 2115 articles were published from 2015 and 2019 on the “antibiotic resistance” and “*Escherichia coli*” in the fields of “Agriculture and Biological Sciences” (1376 articles) and “Environmental Science” (739 articles). According to the results of the analysis, *Escherichia coli* was the most studied bacteria in the wastewater environment. As it is known that wastewater treatment plants are considered as a source of releasing the ARB as traditional wastewater treatment plants are not completely removing the ARGs. Therefore, the release of ARB and ARGs from wastewater treatment plants needs to be reduced. After our results, fulfilling this justification should be the first thing that comes to mind. However, there are a few studies on the water we consume and use, such as drinking water, bathing water, and well water. Therefore, the studies of antibiotic resistance and *Escherichia coli* in these environments should be increased by prioritizing human health. The USA, China, and India were found to be the leading countries based on their number of publications. More studies led by leading countries should be supported and combined for this purpose.

One of the unique values of this study is to offer an innovative solution proposal. Reducing the consumption of antimicrobials is not sufficient to mitigate the release of AMR; therefore, an effective treatment technique is significantly needed. A variety of treatment techniques such as UV-C/H_2_O_2_ and sunlight/H_2_O_2_ (S. G. Michael et al. 2020), ultraviolet disinfection (Wang et al. 2020), oxidative damage (Zhang et al. 2020), membrane filtration and 265 nm UV irradiation (Krzeminski et al. 2020), and UV-activated persulfate (Zhou et al. 2020) have been applied for the removal of antibiotics, antibiotic-resistant *E. coli*, and gene in wastewater. However, there is no particular treatment technique used for their treatment; it is, therefore, very significant to offer an efficient technique to remove them from wastewater. In this study, we accelerate new avenues for using metal-organic frameworks (MOFs) to remove or reduce them effectively from wastewater. MOFs are emerging crystalline hybrid materials that are formed by organic linker buildings and metal ions through coordination bonds (Furukawa et al. 2013). MOFs can be easily tunable and designed to be able to use in a wide range of applications including gas storage and separation (Li et al. 2014), energy storage devices (Baumann et al. 2019), toxic material adsorption (Barea et al. 2014), chemical warfare detoxification (Gil-San-Millan et al. 2017), and biomedical applications (McKinlay et al. 2010; Shen et al. 2020). Moreover, recently, MOFs have been utilized in various antibacterial fields owing to their porosity, tunability, and release capability. Although there are no actual studies on their removal/reduction by MOFs, MOFs are considered as alternative materials for potential techniques. Some studies revealed that MOFs are proven to efficiently use in antibacterial materials for environmental, biological and food antimicrobial (Shen et al. 2020) and antibacterial substances can be captured in MOFs. Therefore, we can give an inspiration to the future studies of the antimicrobial resistance removal via adsorption using MOFs as adsorbents.

Furthermore, an outbreak of novel coronavirus started in 2019 and spread very quickly throughout the world. As a result of this rapid spread and the COVID-19 pandemic, coronavirus also found in sewage in different countries, so it will become vital to offer an efficient solutions to be removing or tracing COVID-19 in the wastewater due to its long-term effect on human health. In general conclusion of this study, permanent solutions of the microbial problems can only be achieved by working under the umbrella of “One Health” which is considering the human-food-environment triangle.

## References

[CR1] Baquero F, Martinez JL, Canton R (2008). Antibiotics and antibiotic resistance in water environments. Current Opinion in Biotechnology.

[CR2] Barea E, Montoro C, Navarro JAR (2014). Toxic gas removal–metal–organic frameworks for the capture and degradation of toxic gases and vapours. Chemical Society Reviews.

[CR3] Baumann AE, Burns DA, Liu B, Thoi VS (2019). Metal-organic framework functionalization and design strategies for advanced electrochemical energy storage devices. Communications Chemistry.

[CR4] Ellegaard O, Wallin JA (2015). The bibliometric analysis of scholarly production: How great is the impact?. Scientometrics.

[CR5] Furukawa H, Cordova KE, O'Keeffe M, Yaghi OM (2013). The chemistry and applications of metal-organic frameworks. Science.

[CR6] Gil-San-Millan R, López-Maya E, Hall M, Padial NM, Peterson GW, DeCoste JB, Rodríguez-Albelo LM, Oltra JE, Barea E, Navarro JAR (2017). Chemical warfare agents detoxification properties of zirconium metal–organic frameworks by synergistic incorporation of Nucleophilic and basic sites. ACS Applied Materials & Interfaces.

[CR7] Hembach N, Alexander J, Hiller C, Wieland A, Schwartz T (2019). Dissemination prevention of antibiotic resistant and facultative pathogenic bacteria by ultrafiltration and ozone treatment at an urban wastewater treatment plant. Scientific Reports.

[CR8] Krzeminski, P., Feys, E., Anglès d'Auriac, M., Wennberg, A. C., Umar, M., Schwermer, C. U., et al. (2020). Combined membrane filtration and 265 nm UV irradiation for effective removal of cell free antibiotic resistance genes from feed water and concentrate. *Journal of Membrane Science, 598*. 10.1016/j.memsci.2019.117676.

[CR9] Li B, Wen H-M, Zhou W, Chen B (2014). Porous metal–organic frameworks for gas storage and separation: What, how, and why?. The Journal of Physical Chemistry Letters.

[CR10] McKinlay AC, Morris RE, Horcajada P, Férey G, Gref R, Couvreur P (2010). BioMOFs: Metal–organic frameworks for biological and medical applications. Angewandte Chemie International Edition.

[CR11] Michael I, Rizzo L, McArdell CS, Manaia CM, Merlin C, Schwartz T, Dagot C, Fatta-Kassinos D (2013). Urban wastewater treatment plants as hotspots for the release of antibiotics in the environment: A review. Water Research.

[CR12] Michael SG, Michael-Kordatou I, Nahim-Granados S, Polo-López MI, Rocha J, Martínez-Piernas AB, Fernández-Ibáñez P, Agüera A, Manaia CM, Fatta-Kassinos D (2020). Investigating the impact of UV-C/H2O2 and sunlight/H2O2 on the removal of antibiotics, antibiotic resistance determinants and toxicity present in urban wastewater. Chemical Engineering Journal.

[CR13] Odonkor ST, Ampofo JK (2013). Escherichia coli as an indicator of bacteriological quality of water: An overview. Microbiology Research.

[CR14] Price RG, Wildeboer D (2017) E. coli as an Indicator of contamination and health risk in environmental waters. In Escherichia coli - Recent Advances on Physiology, Pathogenesis and Biotechnological Applications

[CR15] Rizzo L, Manaia C, Merlin C, Schwartz T, Dagot C, Ploy MC, Michael I, Fatta-Kassinos D (2013). Urban wastewater treatment plants as hotspots for antibiotic resistant bacteria and genes spread into the environment: A review. Sci Total Environ.

[CR16] Shen M, Forghani F, Kong X, Liu D, Ye X, Chen S, Ding T (2020). Antibacterial applications of metal–organic frameworks and their composites. Comprehensive Reviews in Food Science and Food Safety.

[CR17] Thakali, O., Brooks, J. P., Shahin, S., Sherchan, S. P., & Haramoto, E. (2020). Removal of antibiotic resistance genes at two conventional wastewater treatment plants of Louisiana, USA. *Water, 12*(6). 10.3390/w12061729.

[CR18] United Nations (2016) Draft political declaration of the high-level meeting of the General Assembly on antimicrobial resistance. http://go.nature.com/2e3bMdF. Accessed 05 August 2020

[CR19] Voigt AM, Ciorba P, Dohla M, Exner M, Felder C, Lenz-Plet F (2020). The investigation of antibiotic residues, antibiotic resistance genes and antibiotic-resistant organisms in a drinking water reservoir system in Germany. International Journal of Hygiene and Environmental Health.

[CR20] Wang J, Sui M, Li H, Yuan B (2020). The effects of ultraviolet disinfection on vancomycin-resistant enterococcus faecalis. Environmental Science. Processes & Impacts.

[CR21] World Health Organization (2015) Global action plan on antimicrobial resistance. https://www.who.int/antimicrobial-resistance/global-action-plan/en/. Accessed 05 August 2020

[CR22] Zhang L, Jin H, Ma H, Gregory K, Qi Z, Wang C, Wu W, Cang D, Li Z (2020). Oxidative damage of antibiotic resistant E. coli and gene in a novel sulfidated micron zero-valent activated persulfate system. Chemical Engineering Journal.

[CR23] Zhou CS, Wu JW, Dong LL, Liu BF, Xing DF, Yang SS (2020). Removal of antibiotic resistant bacteria and antibiotic resistance genes in wastewater effluent by UV-activated persulfate. Journal of Hazardous Materials.

